# Supplementary material for: The leukemic oncogene EVI1 hijacks a MYC super-enhancer by CTCF-facilitated loops
Source: Nat Commun. 2021 Sep 28;12:5679. doi: 10.1038/s41467-021-25862-3 (PMC8479123; doi:10.1038/s41467-021-25862-3)
Supplement: Supplementary file 1 — Supplementary Information [file 41467_2021_25862_MOESM1_ESM.pdf]

SUPPLEMENTARY INFORMATION

The leukemic oncogene *EVI1* hijacks a *MYC* super-enhancer by CTCF-facilitated loops

Sophie Ottema[1,2,*], Roger Mulet-Lazaro[1,2,*], Claudia Erpelinck-Verschueren[1,2,*], Stanley van Herk[1,2], Marije Havermans[1,2], Andrea Arricibita Varea[1,2], Michael Vermeulen[1], H. Berna Beverloo[3], Stefan Gröschel[4,5], Torsten Haferlach[6], Claudia Haferlach[6], Bas Wouters[1,2], Eric Bindels[1], Leonie Smeenk[1,2,**], and Ruud Delwel[1,2,**]

1 Department of Hematology, Erasmus University Medical Center, Rotterdam, The Netherlands

2 Oncode Institute, Erasmus University Medical Center, Rotterdam, The Netherlands

3 Department of Clinical Genetics, Erasmus University Medical Center, Rotterdam, The Netherlands

4 German Cancer Research Center, A380, Heidelberg, Germany

5 Department of Internal Medicine V, Heidelberg University Hospital, Heidelberg, Germany

6 Munich Leukemia Laboratory, Munich, Germany

* These authors contributed equally

** These authors jointly supervised this work

Correspondence: h.delwel@erasmusmc.nl

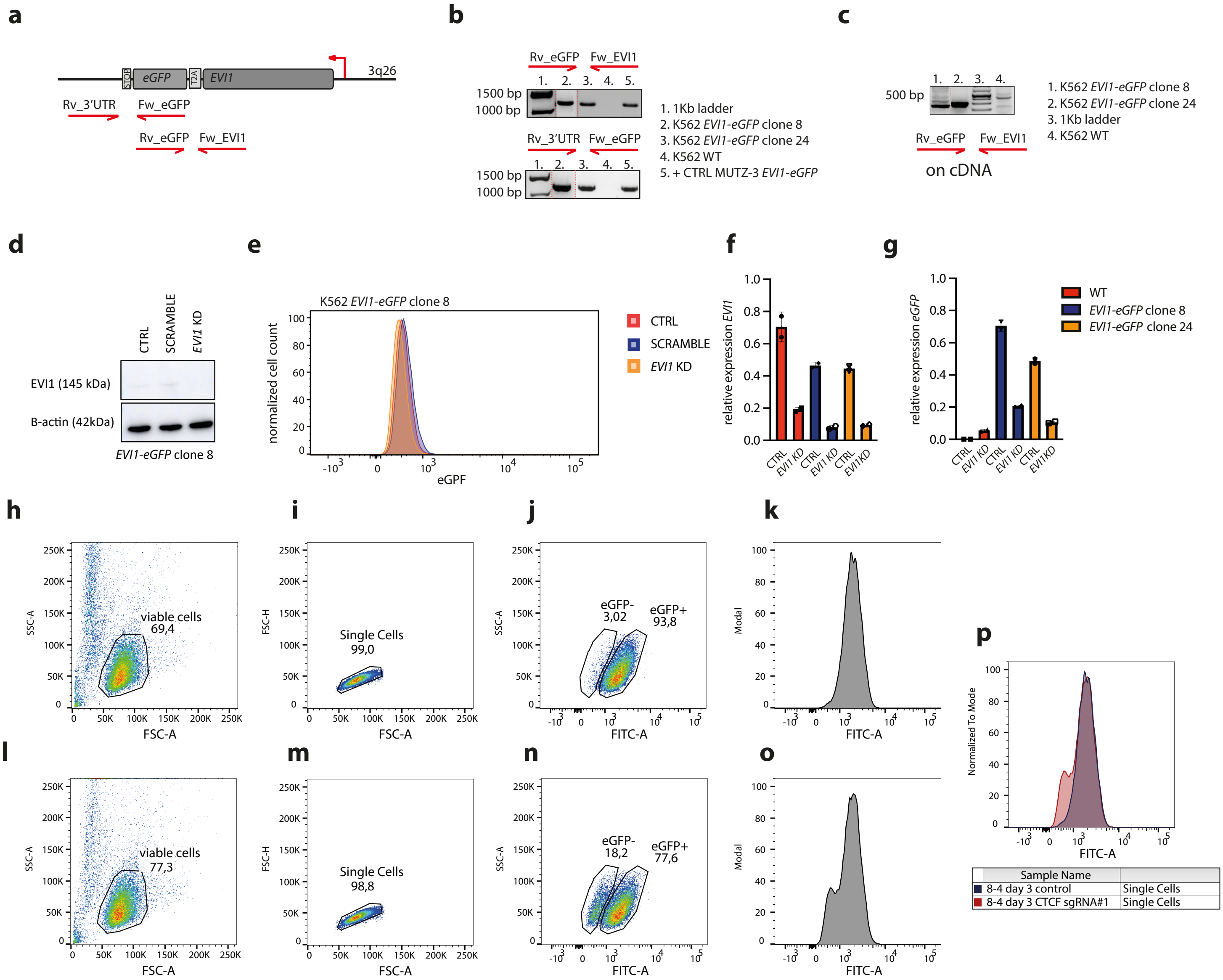

Supplementary Figure 1. Generation of cell model with eGFP reporter for EVI1 expression

(a) Schematic overview of PCR primers located in the eGFP-T2A-EVI1 insert. **(b)** PCRs of genomic DNA of K562 EVI1-eGFP clones to verify correct genotype. Source data are provided as a Source Data file. **(c)** PCR on cDNA to verify EVI1-eGFP transcript in the same clones. Source data are provided as a Source Data file. **(d)** Western blot showing very low EVI1 protein levels in the controls as expected, and absent EVI1 protein levels upon EVI1 knockdown by shRNA. Source data are provided as a Source Data file. **(e)** Flow cytometry plot showing eGFP reduction upon EVI1 knockdown. **(f)** EVI1 expression levels relative to PBGD by qPCR upon EVI1 knockdown in two different K562 EVI1-eGFP clones (clones 8 and 24) and WT K562. Statistical test: one-way ANOVA. The error bar represents the standard deviation (SD). **(g)** eGFP expression levels relative to PBGD by qPCR upon EVI1 knockdown in two different K562 EVI1-eGFP clones (clones 8 and 24) and WT K562. Statistical test: one-way ANOVA. The error bar represents the standard deviation (SD). **(h)** Sorting strategy flow cytometry and FACS sort experiments. A control is shown as an example (clone 8-4, no CRISPR-Cas9 targeting). Gate selects the viable cells. **(i)** Similar to h. Gate selects the single cells. (j) Similar to h. Gate selects the eGFP negative and eGFP positive cells. **(k)** Similar to h. Histogram shows eGFP levels in the FITC-A channel. **(l)** Sorting strategy flow cytometry and FACS sort experiments. An experiment targeting the CTCF binding site near the EVI1 promoter is shown as an example (clone 8-4 CRISPR-Cas9 targeted with CTCF sgRNA#1). Gate selects the viable cells. **(m)** Similar to l. Gate selects the single cells. **(n)** Similar to l. Gate selects the eGFP negative and eGFP positive cells. These gates were used for FACS sorting experiments. Figures 4c, 4d, 4e, 4g, 4h, 5c-f, 6c-g and Supplementary Figures S3a, S5a-c. **(o)** Similar to l. Histogram shows eGFP levels in the FITC-A channel. **(p)** An overlay of the eGFP levels of the control andCRISPR-Cas9 targeted experiments is shown displaying eGFP levels as histograms. This gating and display strategy was used for Figures 2b, 2d, 2g, 2h, 4b, 5b, 6b and Supplementary Figures S1e, S2g, S4a, S4g and S7a.

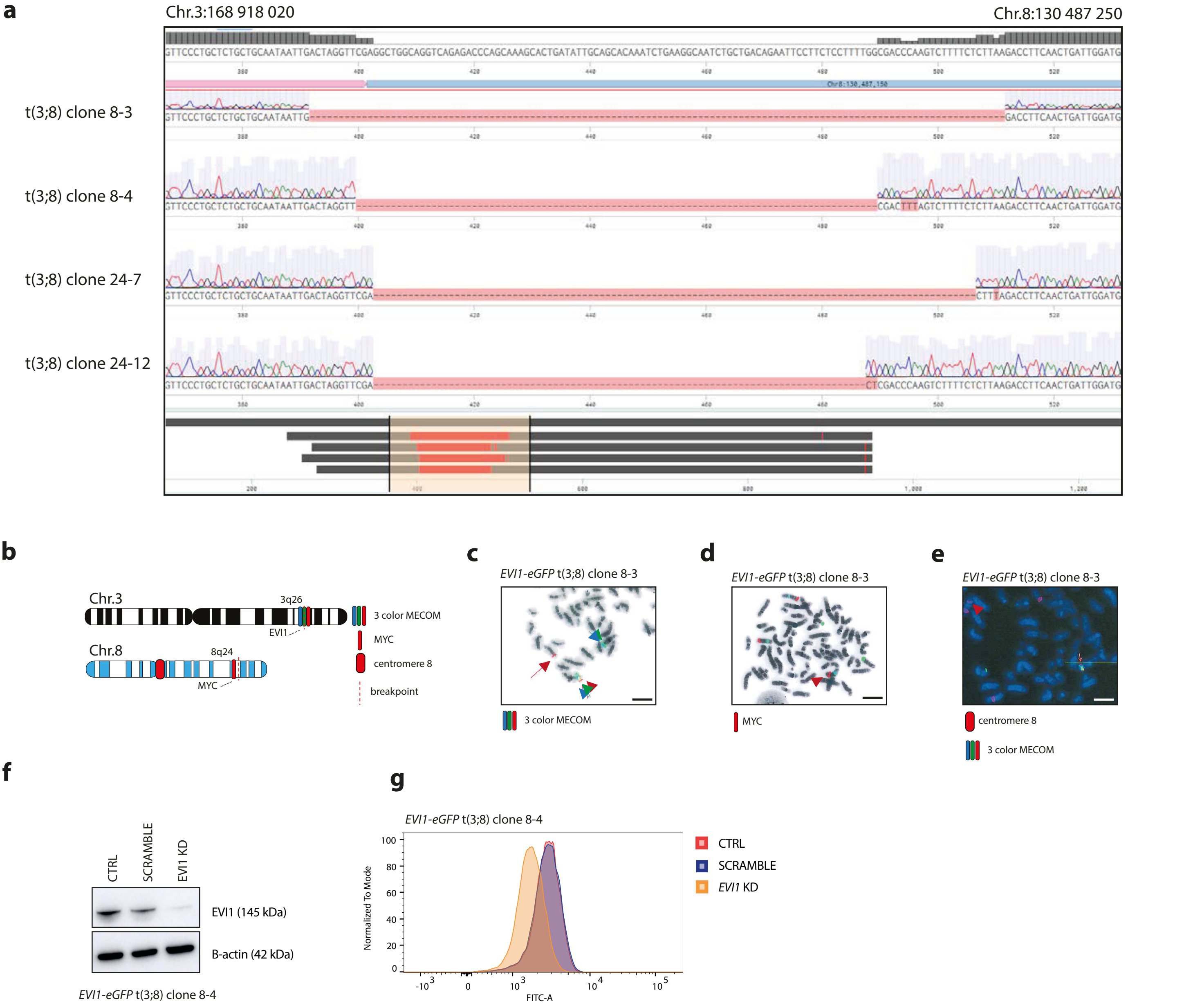

Supplementary Figure 2. A t(3;8) cell model recapitulates EVI1 overexpression in human AML

(a) Sanger sequencing (amplicon covering the breakpoint as shown in Figure 2G), to validate the generation of a t(3;8). Nucleotide sequencing data of all four K562 EVI1-eGFP t(3;8) clones is shown. Using the online tool BLAT 1 the left part of the sequence maps back to 3q26.2 (pink) and the right part maps to 8q24 (blue). About 100bp were deleted on the Chr.8 side of the breakpoint in the generation of the translocation (depicted below in red). **(b)** Schematic overview of diagnostic FISH experiments performed to validate the presence of t(3;8) in the four clones. Fluorescent FISH pictures of K562 t(3;8) clone 8-3 are in C-E are shown. **(c)** Detection of t(3;8) by FISH. MECOM was split and the red signal was separated from the blue and green, indicating a translocation of the distal part of the q arm of chromosome 3. Scale bar 5 µm. **(d)** Fluorescent images obtained by FISH to detect t(3;8). Three red signals represent the MYC gene on chromosomes 8 (3 copies of Chr.8 in K562), demonstrating that the MYC gene is unaffected. The longer tip of the derivate Chr.8 near the MYC signal (arrow) is in line with a (t(3;8)) rearrangement at this chromosome. Scale bar 5 µm. **(e)** Fluorescent images obtained by FISH to detect t(3;8). The three Chr.8s can be visualized with the bigger red signal at the centromeres; the red part of the separated MECOM probe is located at the q-arm of one of the Chr.8s (arrow). Together the FISH images demonstrate that part of MECOM had been translocated to Chr.8, forming a t(3;8)(q26;q24). Scale bar 5 µm. **(f)** Western blot showing high EVI1 protein levels for t(3;8) clone 8-4, and a reduction of EVI1 levels upon EVI1 knockdown (KD) using a shRNA. Source data are provided as a Source Data file. **(g)** Flow cytometry plot showing eGFP reduction upon EVI1 knockdown.

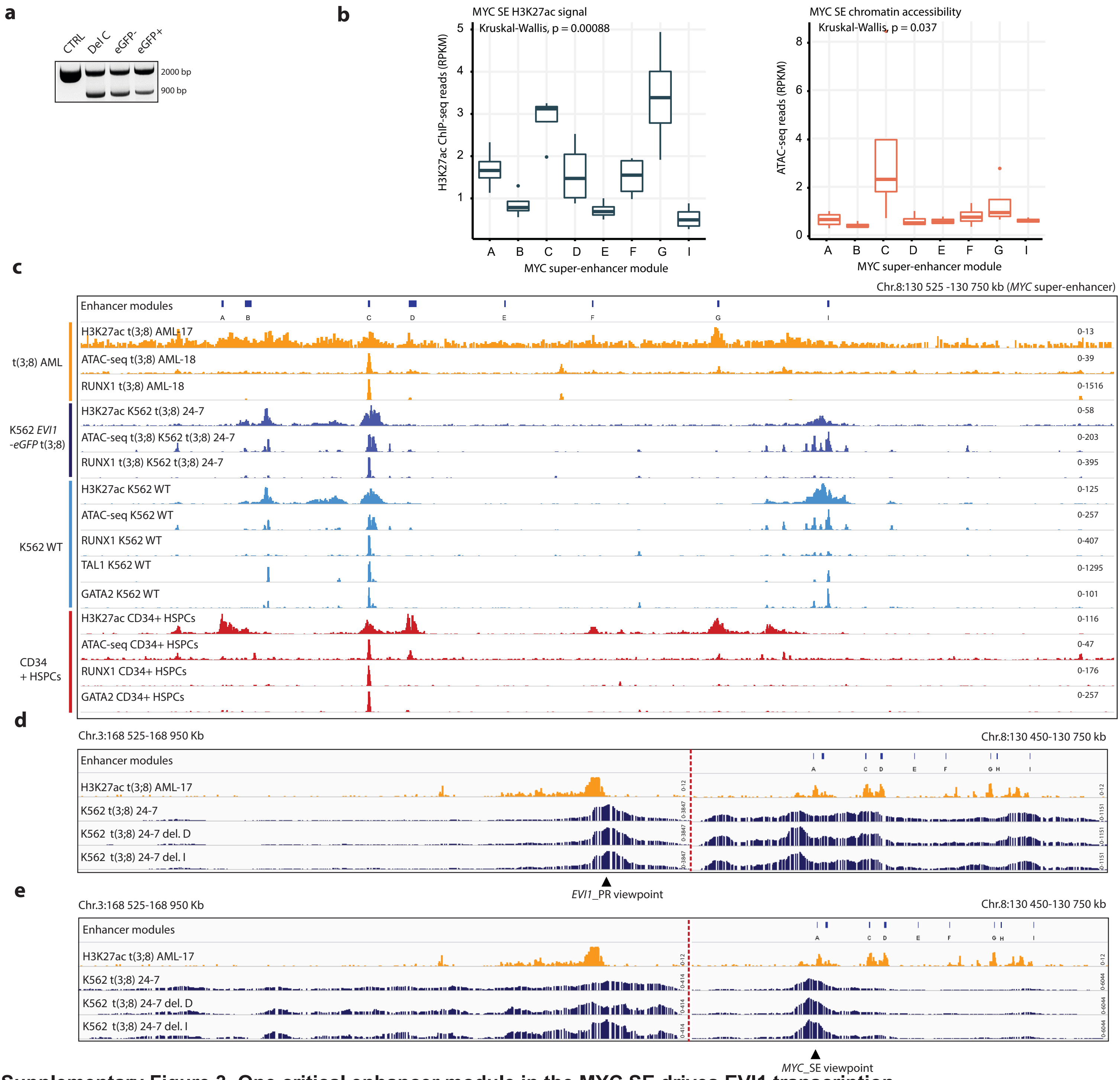

Supplementary Figure 3. One critical enhancer module in the MYC SE drives EVI1 transcription

(a) PCR showing the deletion of enhancer module C present in the bulk cells, eGFP- and eGFP+ fractions, but not in the controls. The deletion observed by the smaller PCR band in the eGFP+ fraction can be explained by deletions in the non-translocated Chr.8 allele not influencing EVI1 expression, and thus not eGFP. Nevertheless, clearly more cells with the deletion are found in the eGFP- fraction. Source data are provided as a Source Data file. **(b)** Boxplots depicting the accessibility and activity of the MYC SE modules as measured by ATAC-seq (N=5) and H3K27ac ChIP-seq (N=4) in t(3;8) AML patients. The Y axis indicates the Reads Per Kilobase of transcript per Million mapped reads (RPKM) for each module. Statistically significant differences were determined by a Kruskal-Wallis test. Both ATAC-seq and H3K27ac showed that module C is distinctly active. The lower and upper edges of the boxplots represent the first and third quartiles, respectively, the horizontal line inside the box indicates the median. The whiskers extend to the most extreme values within the range comprised between the median and 1.5 times the interquartile range. The circles represent outliers outside this range. **(c)** MYC SE module C is active and bound by HSPC-active transcription factors in t(3;8) AML (orange tracks), t(3;8) K562 (dark blue), wild type K562 (light blue) and CD34+ cells (red). For each of these groups, ATAC-seq, H3K27ac ChIP-seq and transcription factor ChIP-seq are shown. Transcription factor ChIP-seq data for CD34+ and wild type K562 cells are publicly available from 2 and 3 respectively. **(d)** 4C-seq data of t(3;8) clone 24-7 (blue tracks) with the EVI1 promoter as viewpoint (black triangle). No interaction changes were observed following the deletions of MYC SE modules D or I (Figure 4B). The top H3K27ac ChIP-seq track (orange) shows the presence of the active EVI1 promoter and the modules of the MYC SE. **(e)** 4C-seq data of t(3;8) clone 24-7 (blue tracks) with viewpoint in the MYC SE (black triangle) showing the same as D. No interaction changes upon deletion of MYC SE modules D or I were observed (Figure 4B).

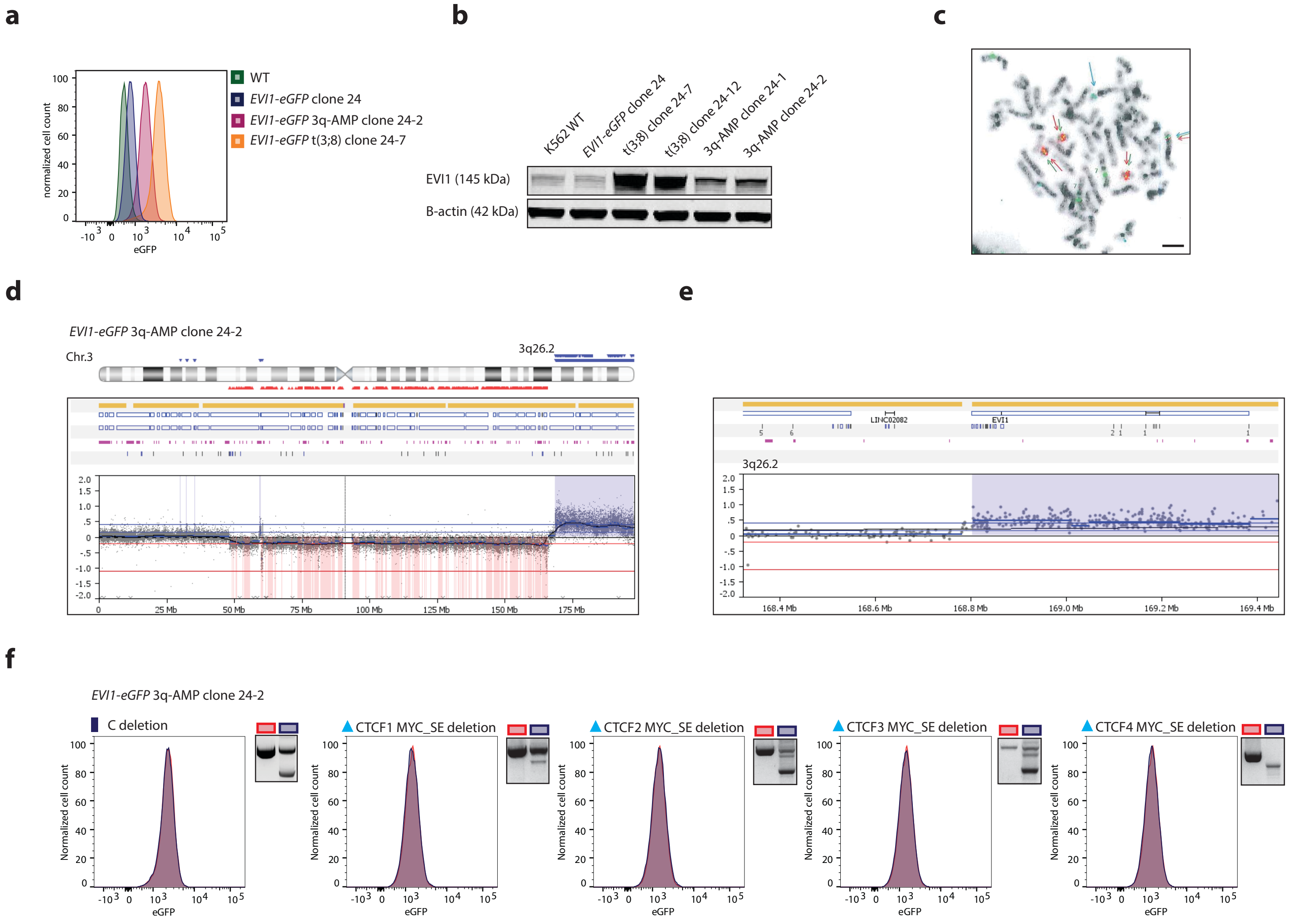

Supplementary Figure 4. EVI1 is not regulated by MYC SE in a control model with 3q/MECOM amplification

(a) Flow cytometry plot comparing eGFP levels of EVI1-eGFP K562 with 3q/MECOM amplification (EVI1-AMP) clone 24-2 to WT, parental (EVI1-eGFP) and t(3;8) clone 24-7 K562 cells. **(b)** Western blot comparing EVI1 protein levels of EVI1-AMP clones 24-1 and 24-2 to WT, parental (EVI1-eGFP) and t(3;8) clones 24-7 and 24-12. Source data are provided as a Source Data file. **(c)** MECOM (EVI1) FISH for clone 24-2 illustrating the EVI1 amplification. Scale bar 5 µm. **(d)** Overview of Chr.3 SNP array data showing high copy number for the q arm of Chr.3 starting from 3q26.2 in clone 24-2. Copy number gains are indicated in blue, whereas copy number losses are indicated in red. **(e)** SNP array data: zoom-in on the 3q26.2 locus of clone 24-2 indicating the breakpoint/start of the amplification (blue area). The amplification includes exactly the complete EVI1 (MECOM) locus. We estimated clone 24-2 has 4 copies of this part of the 3q arm including EVI1, resulting in elevated EVI1 expression and protein levels. **(f)** Clone 24-2 was used as a control clone with high EVI1 expression but without a t(3;8). All genomic deletions in the MYC SE (Figure 4A and 5A) made in this study were also successfully performed in clone 24-2 as shown here by PCR. Flow analysis showed that none of the deletions produced changes in EVI1 expression in this clone.

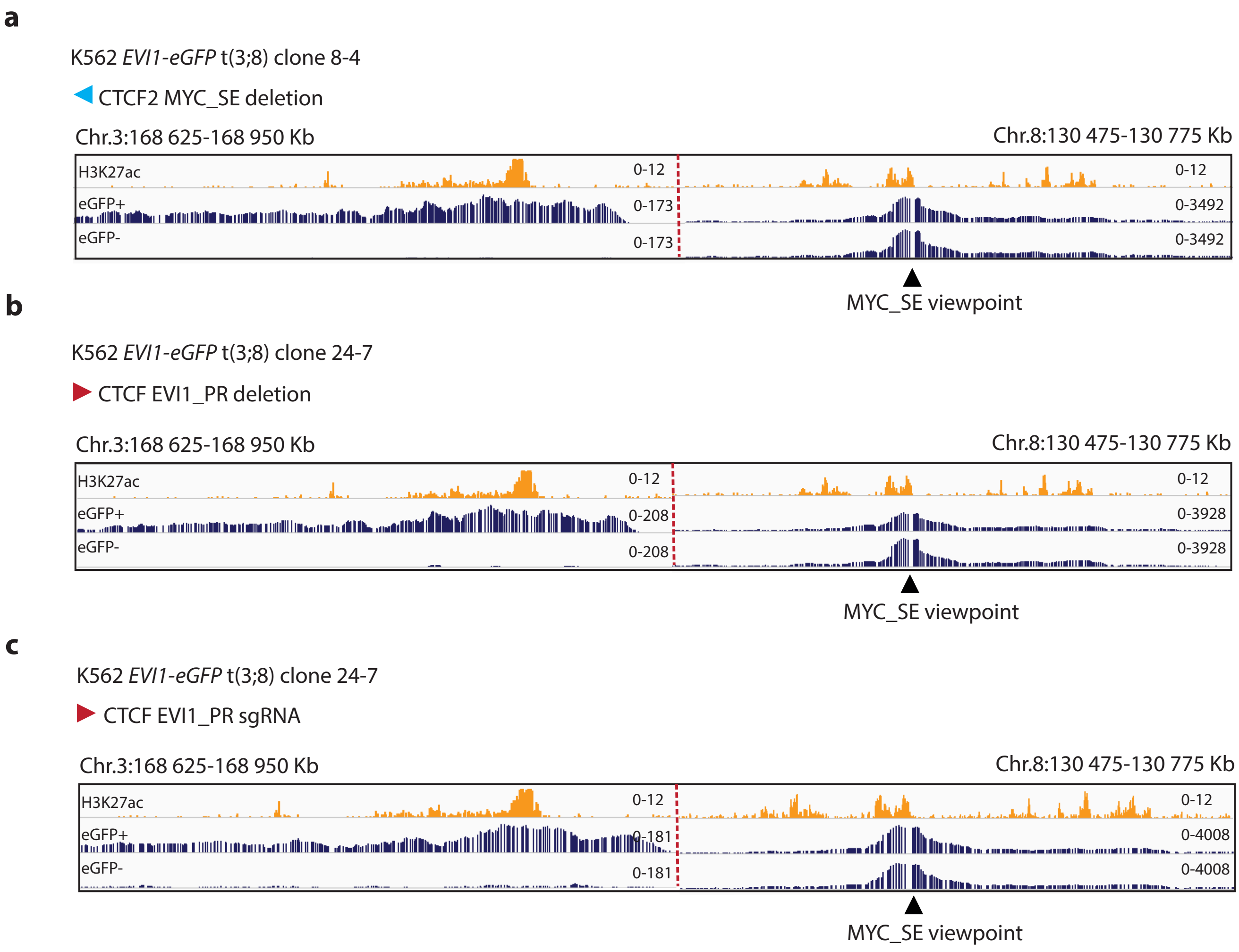

Supplementary Figure 5. CTCF binding site upstream of the EVI1 promoter hijacks a MYC SE in t(3;8) AML
(a) 4C-seq data (t(3;8) clone 8-4), with the MYC SE as viewpoint, of cells with a CTCF2 MYC SE deletion and sorted on eGFP. In the eGFP- fraction (lower blue), a loss of interaction with the EVI1 promoter was observed compared to the eGFP+ cells (upper blue). The top H3K27ac ChIP-seq track (orange) shows the presence of the active EVI1 promoter and the modules of the MYC SE. **(b)** 4C-seq data (t(3;8) clone 24-7), with the MYC SE as viewpoint, of cells with a CTCF EVI1 promoter deletion and sorted on eGFP. In the eGFP- fraction (lower blue), a loss of interaction with the EVI1 promoter was observed compared to the eGFP+ cells (upper blue). The top H3K27ac ChIP-seq track (orange) shows the presence of the active EVI1 promoter and the modules of the MYC SE. **(c)** Comparison of chromatin interaction at the EVI1 promoter for eGFP+ (upper blue) and eGFP- (lower blue) cells, shown by 4C-seq (t(3;8) clone 24-7) with the MYC SE as viewpoint after targeting the CTCF motif with the sgRNA as presented in Figure 6D.

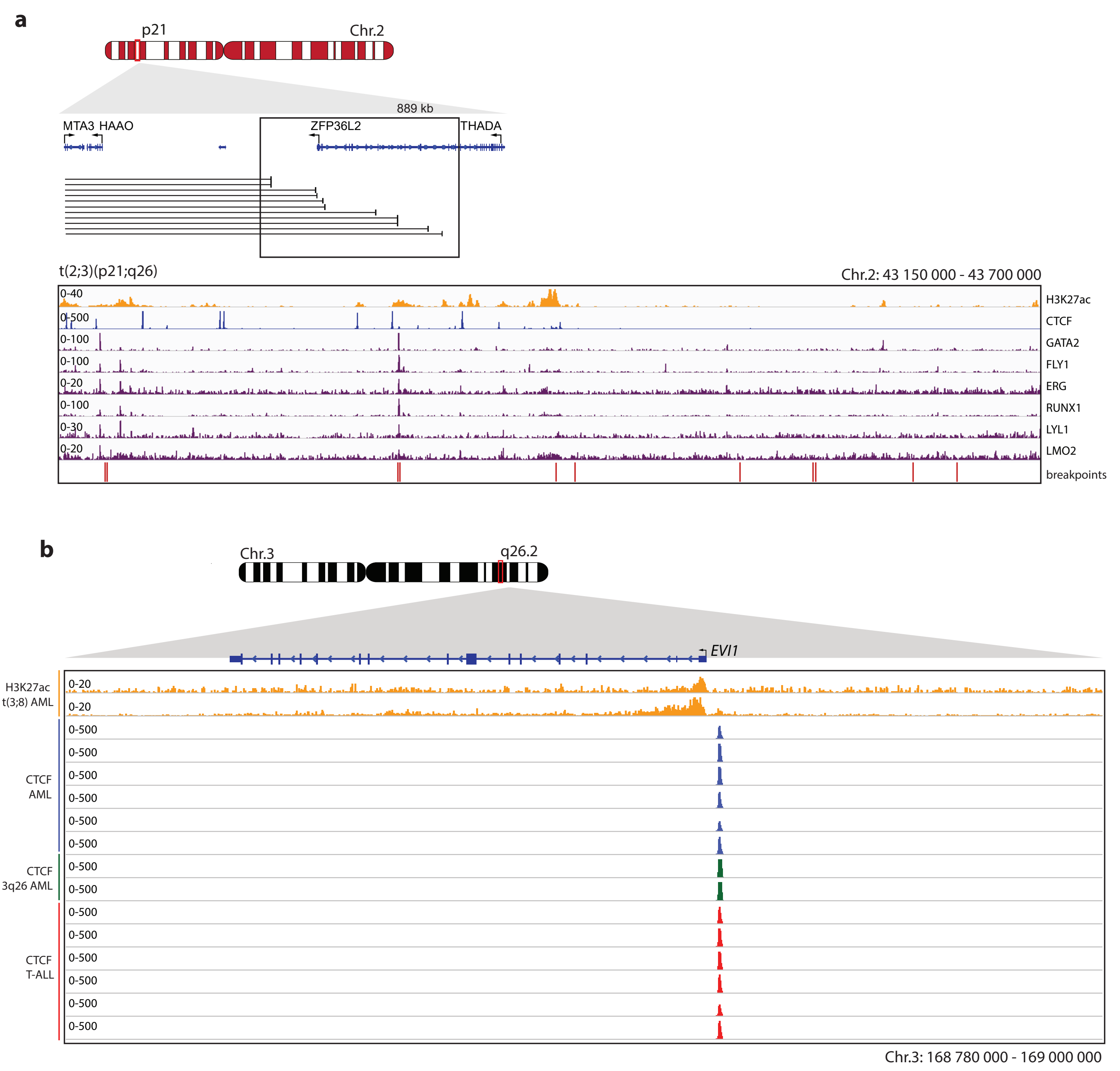

Supplementary Figure 6. Translocated region in t(2;3)(p21;q26) AML contains strong regulatory elements

(a) Translocated locus in t(2;3)(p21;q26) AML showing the presence of the gene THADA, the black box indicating the zoom-in shown below. Zoom-in: ChIP-seq for H3K27ac (t(3;8) patient AML-17, orange) indicating putative enhancer regions, CTCF binding and HSPC-active transcription factor recruitment. The red lines below indicate the exact breakpoint of the t(2;3) AMLs (detected by 3q-seq). We predict that the translocated region of each of these cases could be unique, but in all cases contains a strong regulatory locus. **(b)** ChIP-seq data showing H3K27ac in 2 t(3;8) AML patients (yellow), CTCF binding in 6 non-3q26 AML cases (blue), 2 AML cases with 3q26 rearrangements (green) and in 6 T-ALL cases (red). EVI1 is only expressed in the 3q26-rearranged AML cases (TPM > 1), comprising one t(3;8) and one inv(3) patient. Thus, CTCF binding seems to be constitutive and independent of EVI1 expression.

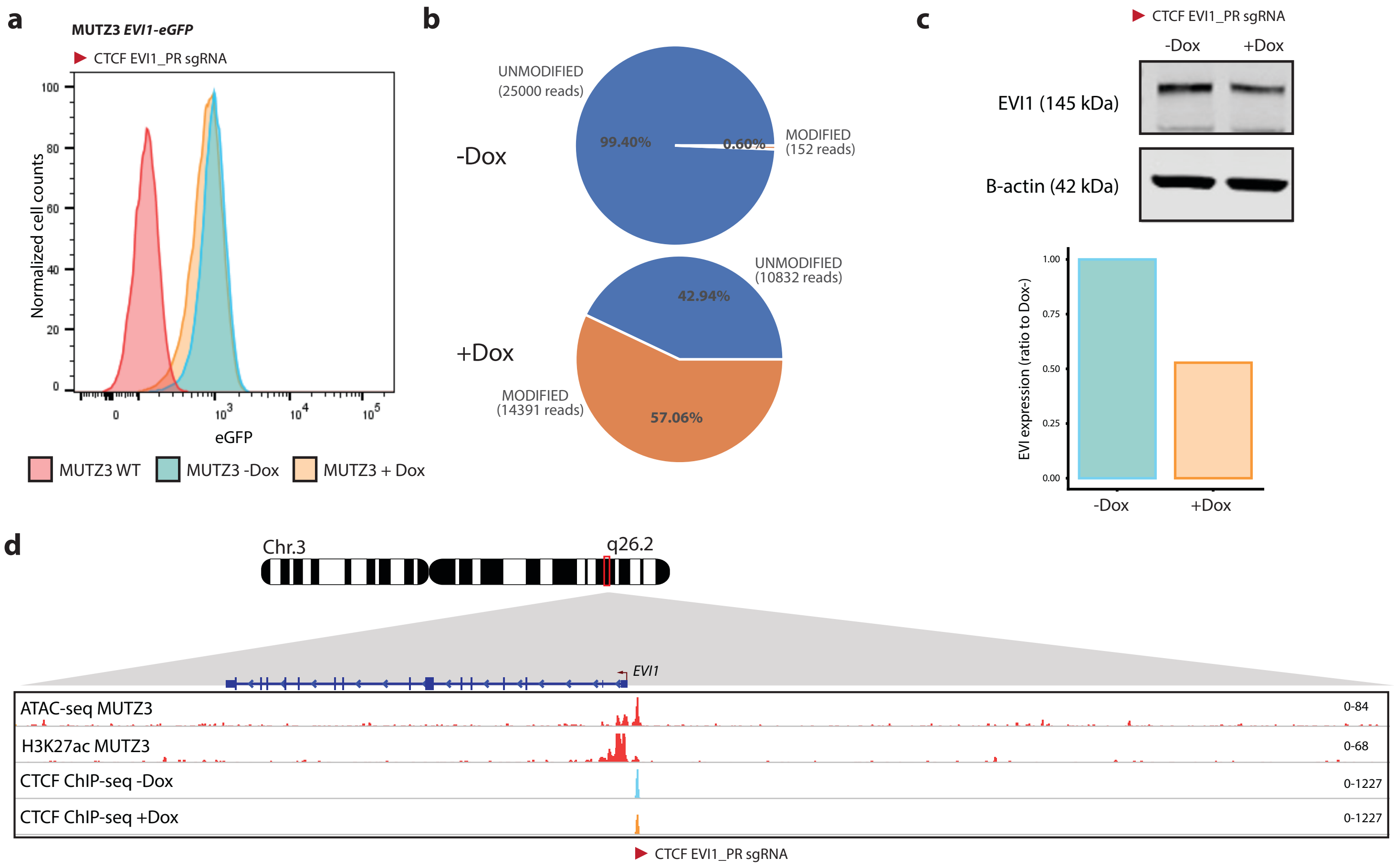

Supplementary Figure 7. The CTCF binding site upstream of EVI1 is also critical for enhancer hijacking in other models of 3q26-rearranged AML

(a) Flow cytometry plot showing the effect of targeting the CTCF EVI1_PR site in MUTZ3 cells with sgRNA. Cas9 was induced by doxycycline (Dox), leading to loss of eGFP (orange) compared to control cells (blue). In red, wild type MUTZ3 cells without the eGFP reporter. **(b)** Amplicon-seq data showing the percentage of modified (orange) and unmodified (blue) reads in MUTZ3 after targeting the EVI1_PR CTCF with sgRNA. Cas9 was induced by doxycycline (+Dox), leading to successful genome editing compared to control cells (-Dox). **(c)** Western blot showing depletion of EVI1 protein in MUTZ3 after targeting the CTCF EVI1_PR binding site with sgRNA. Source data are provided as a Source Data file. The barplot below presents the expression levels of EVI1 measured as EVI1/B-actin ratio, relative to expression in Dox- cells. The bars only represent one data point. **(d)** Epigenomic landscape of the EVI1 promoter in MUTZ3 following the deletion of the CTCF EVI1_PR site. In red, ATAC-seq and H3K27ac ChIP-seq from wild type MUTZ3 cells indicating the presence of an active promoter. CTCF ChIP-seq shows a moderate loss of CTCF upon targeting of the CTCF EVI1_PR site with sgRNA and Cas9 induction (orange), compared to control cells without Cas9 induction (blue).

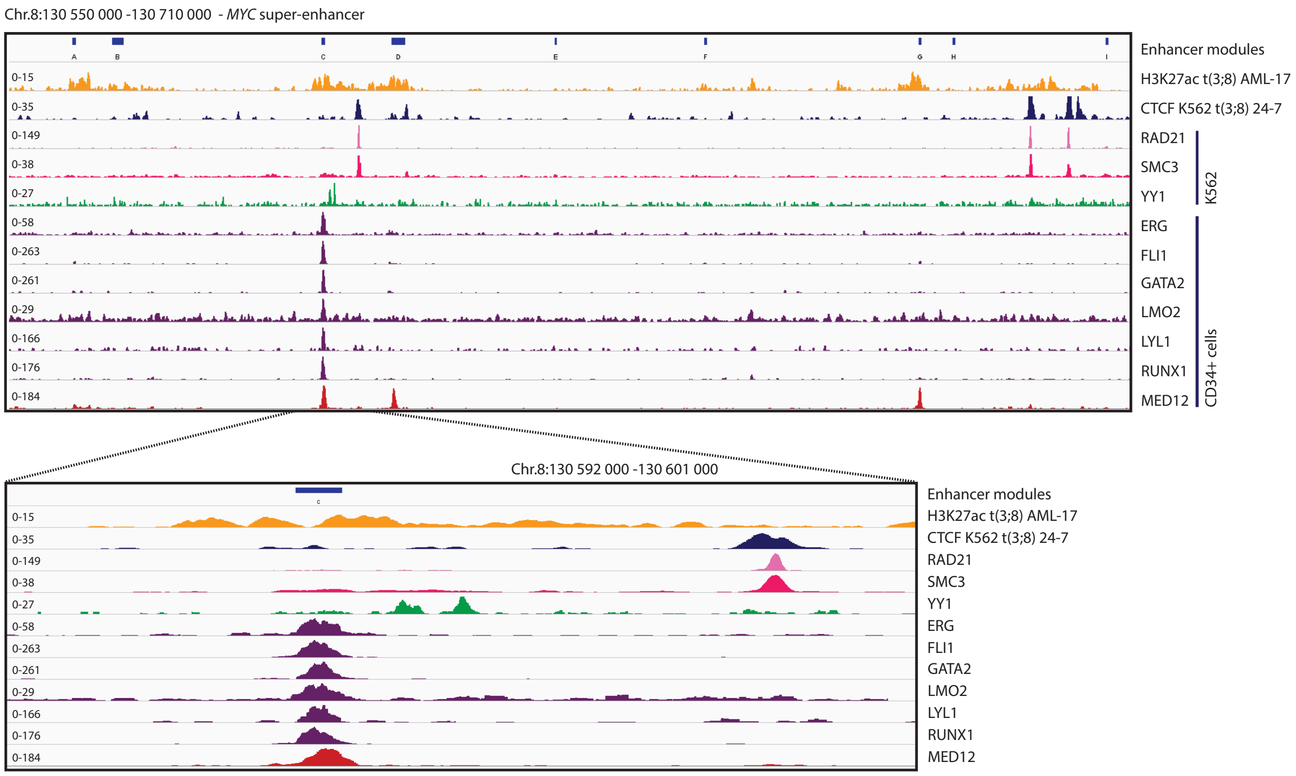

Supplementary Figure 8. Transcription factor and transcriptional co-factor occupation of the MYC SE
In the upper panel, the MYC SE and its distinct enhancer modules are illustrated; in the lower panel, a zoom-in on enhancer module C is shown. Active enhancer regions are illustrated by H3K27ac of the t(3;8) AML-17 patient (orange) and CTCF binding locations are marked by CTCF ChIP-seq data of t(3;8) K562 clone 24-7. ChIP-seq data in K562 WT of cohesin subunits RAD21 (light pink) and SMC2 (pink), as well as YY1 (green), were retrieved from ENCODE 4. ChIP-seq data in CD34+ from healthy donors were retrieved from other publications, including a heptad of hematopoietic transcription factors 3 (ERG, FLI1, GATA2, LMO2, LYL1, and RUNX1) in purple and MED12 5 in red.

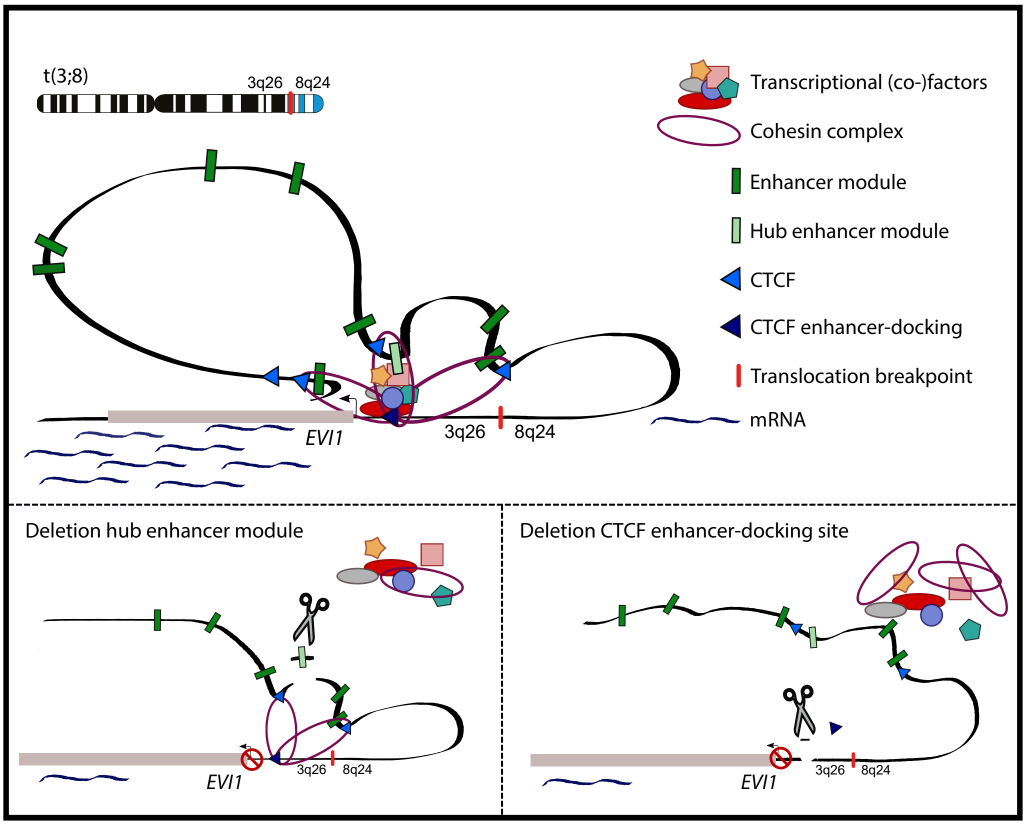

Supplementary Figure 9. Interaction between the EVI1 promoter and the MYC SE in t(3;8) AML

In the upper panel: proposed model for the regulation of EVI1 expression by the MYC SE.The MYC SE is organized as a series of modules that interact with the EVI1 promoter via CTCF-facilitated loops, leading to the overexpression of EVI1. Module C, which is bound by hematopoietic transcription factors, plays a critical role in the organization of the MYC super-enhancer as a hub enhancer. In the bottom left panel: deletion of module C (scissors indicate cleavage locations) prevents recruitment of transcription factors and results in the downregulation of EVI1. In the bottom right panel: deletion of the CTCF binding site upstream of EVI1 prevents interaction with the MYC SE, leading to downregulation of EVI1

Supplementary Tables

Supplementary Table 1. Materials, resources and software used in this study

REAGENT or RESOURCE	SOURCE	IDENTIFIER
**Antibodies**		
EVI1	Cell Signaling	#2256, #2265
B-Actin	Sigma	clone AC15, #A5441
GAPDH	Santa Cruz	#A5441
CAS9	Biolegend	clone 7A9, #7A9
H3K27ac	Diagenode	#C15410196
H3K9ac	Diagenode	#C15410004
H3K4me3	Diagenode	#C15410003
RUNX1	Abcam	Ab23980
CTCF	Cell signalling	2899S
**Biological Samples**		
Total RNA isolated using Trizol	Thermo Scientific	15596018
Whole cell / protein lysates	Custom buffer	NA
Nuclear / protein lysates	Thermo Scientific	78833
Genomic DNA	Thermo Scientific	K1820-01
ChIP DNA	Custom protocol	NA
**Chemicals, Peptides, and Recombinant Proteins**		
Alt-R S.p. Cas9 Nuclease V3	IDT	1081058
DPNII 5000U	NEB	#R0543M
Csp6I 1500U	Fermentas	#ER0211
TRIzol	Invitrogen	15596018
T4 ligase 5U/ul	Roche	#10799009001
**Critical Commercial Assays**		
Microplex library preparation kit V2	Diagenode	C05010013
KAPA RNA HyperPrep Kit with RiboErase (HMR) Illumina® Platforms	Roche	KK8561 08098140702
Q5® High-Fidelity DNA Polymerase	New England Biolabs	M0491L
KAPA HiFi HotStart Ready mix	Roche	KK2602
KAPA Hyper Prep Kit	Roche	KK8502
TruSeq Custom Amplicon index kit	Illumina	FC-130-1003
Nextera DNA Library Prep Kit	Illumina	FC-121-1030
NEXTFLEX® ChIP-Seq Library Prep Kit for Illumina Sequencing	Perkin Elmer	#NOVA-5143-02
Neon™ Transfection System 100 µL Kit	Thermo Fisher Scientific	MPK10096
AllPrep DNA/RNA Mini kit	Qiagen	80204
Qiaquick PCR purification kit	Qiagen	28104
BigDye™ Terminator v1.1 Cycle Sequencing Kit	Thermo Fisher Scientific	4337450
Fast SYBR™ Green Master Mix	Thermo Fisher Scientific	#4385612
Expand long template PCR system	Roche	#11759060001
**Deposited Data**		
Sequence data generated	This paper	EBI EGA:EGAS00001004808
3q-seq data inv(3)/t(3;3)	[6]	ArrayExpress E-MTAB-2224
ChIP-seq of heptad transcription factors in CD34+ cells	[3]	Gene Expression Omnibus GSE38865

RNA-seq data of hematopoietic stem and progenitor cells	Blueprint[7]	http://blueprint-data.bsc.es/release_2016-08/#!/
RAD21	ENCODE[2,4]	GSM935319
SMC3	ENCODE[2,4]	GSM935310
YY1	ENCODE[2,4]	GSM935368
MED12	[5]	GSM1970177
**Experimental Models: Cell Lines**		
K562	DSMZ	89121407
K562-*EVI1-eGFP*	This paper	NA
t(3;8) AML used for ChIP-seq and 4C-seq karyotype: 46,XX,t(9;22)(q34;q11),t(3;8)(q25?;q24?)	Department of clinical genetics, Erasmus MC, Rotterdam	AML-17
**Oligonucleotides**		
PCR primers Table S2	This paper	NA
qPCR primers Table S2	This paper	NA
Amplicon-seq primers Table S2	This paper	NA
sgRNA sequences Table S2	This paper	NA
**Recombinant DNA**		
dCAS9-VP64_2A_GFP	Addgene	#61422
pX330-U6-Chimeric_BB-CBh-hSpCas9	Addgene	#42230
pCW-Cas9	Addgene	#50661
pDonor for EVI1-eGFP	Smeenk et al., 2021[8]	NA
**Software and Algorithms**		
Prism 8	GraphPad	https://www.graphpad.com/
Image studio lite V5.2	LI-COR	https://www.licor.com
Illustrator	Adobe	https://www.adobe.com/
R v4.0.0	R Core Team	https://www.R-project.org
RStudio v1.3	RStudio	https://rstudio.com/
FlowJo v10.0	BD Biosciences	https://www.flowjo.com/
FACS Diva v9.0	BD Biosciences	https://www.bdbiosciences.com/en-nl/products/software/instrument-software/bd-facsdiva-software
bcl2fastq v2	NA	https://support.illumina.com/downloads/bcl2fastq-conversion-software-v2-20.html
Bowtie v1.1.1	Langmead et al., 2009 [9]	https://sourceforge.net/projects/bowtie-bio/files/bowtie/1.3.0/
bowtie2 v2.3.4.1	Langmead and Salzberg, 2012 [10]	https://sourceforge.net/projects/bowtie-bio/files/bowtie2/2.4.1/
DeepTools bamcoverage v3.43	Ramirez et al., 2016 [11]	https://github.com/deeptools/deepTools
MACS2 v2.2.7.1	Zhang et al., 2008 [12]	https://github.com/macs3-project/MACS
IGV v2.8	Robinson et al., 2011 [13]	http://software.broadinstitute.org/software/igv/

Salmon version 0.13.1	Patro et al., 2017 [14]	https://github.com/COMBINE-lab/salmon/releases
Tximport (R package)	Soneson et al., 2015 [15]	https://bioconductor.org/packages/release/bioc/html/tximport.html
DESeq2 (R package)	Love et al., 2014 [16]	https://bioconductor.org/packages/release/bioc/html/DESeq2.html
TrimGalore v0.4.4	Krueger, 2012 [17]	https://www.bioinformatics.babraham.ac.uk/projects/trim_galore/
BBMap v34.92	Bushnell, 2014 [18]	https://sourceforge.net/projects/bbmap/
CRISPResso2 v2.0.27	Clement et al., 2019 [19]	https://github.com/pinellolab/CRISPResso2
CTCFBSDB 2.0	Ziebarth JD et al., 2013 [20]	https://insulatordb.uthsc.edu/
JASPAR 2020 release	Forners O et al., 2019 [21]	http://jaspar.genereg.net/

Supplementary Data

Supplementary Data 1. Primers and single guide RNAs used in this study

Supplementary References

1 Kent, W. J. BLAT--the BLAST-like alignment tool. *Genome Res* **12**, 656-664 (2002).

2 Davis, C. A. *et al.* The Encyclopedia of DNA elements (ENCODE): data portal update. *Nucleic Acids Res* **46**, D794-D801 (2018).

3 Beck, D. *et al.* Genome-wide analysis of transcriptional regulators in human HSPCs reveals a densely interconnected network of coding and noncoding genes. *Blood* **122**, e12-22 (2013).

4 Encode Project Consortium. An integrated encyclopedia of DNA elements in the human genome. *Nature* **489**, 57-74, doi:10.1038/nature11247 (2012).

5 Aranda-Orgilles, B. *et al.* MED12 Regulates HSC-Specific Enhancers Independently of Mediator Kinase Activity to Control Hematopoiesis. *Cell Stem Cell* **19**, 784-799 (2016).

6 Groschel, S. *et al.* A single oncogenic enhancer rearrangement causes concomitant EVI1 and GATA2 deregulation in leukemia. *Cell* **157**, 369-381 (2014).

7 Fernandez, J. M. *et al.* The BLUEPRINT Data Analysis Portal. *Cell Syst* **3**, 491-495 e495, doi:10.1016/j.cels.2016.10.021 (2016).

8 Smeenk, L. *et al.* Selective requirement of MYB for oncogenic hyperactivation of a translocated enhancer in leukemia. *Cancer Discov*, doi:10.1158/2159-8290.CD-20-1793 (2021).

9 Langmead, B. Aligning short sequencing reads with Bowtie. *Curr Protoc Bioinformatics* **Chapter 11**, Unit 11 17, doi:10.1002/0471250953.bi1107s32 (2010).

10 Langmead, B. & Salzberg, S. L. Fast gapped-read alignment with Bowtie 2. *Nat Methods* **9**, 357-359, doi:10.1038/nmeth.1923 (2012).

11 Ramirez, F. *et al.* deepTools2: a next generation web server for deep-sequencing data analysis. *Nucleic Acids Res* **44**, W160-165, doi:10.1093/nar/gkw257 (2016).

12 Zhang, Y. *et al.* Model-based analysis of ChIP-Seq (MACS). *Genome Biol* **9**, R137-R137, doi:10.1186/gb-2008-9-9-r137 (2008).

13 Robinson, J. T. *et al.* Integrative genomics viewer. *Nat Biotechnol* **29**, 24-26, doi:10.1038/nbt.1754 (2011).

14 Patro, R., Duggal, G., Love, M. I., Irizarry, R. A. & Kingsford, C. Salmon provides fast and bias-aware quantification of transcript expression. *Nat Methods* **14**, 417-419, doi:10.1038/nmeth.4197 (2017).

15 Soneson, C., Love, M. I. & Robinson, M. D. Differential analyses for RNA-seq: transcript-level estimates improve gene-level inferences. *F1000Res* **4**, 1521, doi:10.12688/f1000research.7563.2 (2015).

16 Love, M. I., Huber, W. & Anders, S. Moderated estimation of fold change and dispersion for RNA-seq data with DESeq2. *Genome Biol* **15**, 550, doi:10.1186/s13059-014-0550-8 (2014).

17 Trim Galore (2012).

18 Bushnell, B. BBMap: A Fast, Accurate, Splice-Aware Aligner. (2014).

19 Clement, K. *et al.* CRISPResso2 provides accurate and rapid genome editing sequence analysis. *Nat Biotechnol* **37**, 224-226, doi:10.1038/s41587-019-0032-3

10.1038/s41587-019-0032-3 [pii] (2019).

20 Ziebarth, J. D., Bhattacharya, A. & Cui, Y. CTCFBSDB 2.0: a database for CTCF-binding sites and genome organization. *Nucleic Acids Res* **41**, D188-194, doi:10.1093/nar/gks1165 (2013).

21 Fornes, O. *et al.* JASPAR 2020: update of the open-access database of transcription factor binding profiles. *Nucleic Acids Res* **48**, D87-D92, doi:10.1093/nar/gkz1001 (2020).
