# Supplementary material for: The leukemic oncogene EVI1 hijacks a MYC super-enhancer by CTCF-facilitated loops
Source: Nat Commun. 2021 Sep 28;12:5679. doi: 10.1038/s41467-021-25862-3 (PMC8479123; doi:10.1038/s41467-021-25862-3)
Supplement: Supplementary file 3 — Description of Additional Supplementary Files [file 41467_2021_25862_MOESM3_ESM.pdf]

Description of Additional Supplementary Files

File Name: Supplementary Data 1
Description: Excel file with multiple tabs listing sgRNAs for CRISPR-Cas9 genome editing, qPCR primers, amplicon sequencing primers, PCR primers and 4C primers.
