# Supplementary material for: The leukemic oncogene EVI1 hijacks a MYC super-enhancer by CTCF-facilitated loops
Source: Nat Commun. 2021 Sep 28;12:5679. doi: 10.1038/s41467-021-25862-3 (PMC8479123; doi:10.1038/s41467-021-25862-3)
Supplement: Supplementary file 5 — Reporting Summary [file 41467_2021_25862_MOESM5_ESM.pdf]

Corresponding author(s): Ruud Delwel

Last updated by author(s): Jul 24, 2021

Reporting Summary

Nature Research wishes to improve the reproducibility of the work that we publish. This form provides structure for consistency and transparency in reporting. For further information on Nature Research policies, see our Editorial Policies and the Editorial Policy Checklist.

Statistics

For all statistical analyses, confirm that the following items are present in the figure legend, table legend, main text, or Methods section.

n/a	Confirmed	
☐	☒	The exact sample size (*n*) for each experimental group/condition, given as a discrete number and unit of measurement
☐	☒	A statement on whether measurements were taken from distinct samples or whether the same sample was measured repeatedly
☐	☒	The statistical test(s) used AND whether they are one- or two-sided<br>*Only common tests should be described solely by name; describe more complex techniques in the Methods section.*
☒	☐	A description of all covariates tested
☒	☐	A description of any assumptions or corrections, such as tests of normality and adjustment for multiple comparisons
☐	☒	A full description of the statistical parameters including central tendency (e.g. means) or other basic estimates (e.g. regression coefficient) AND variation (e.g. standard deviation) or associated estimates of uncertainty (e.g. confidence intervals)
☐	☒	For null hypothesis testing, the test statistic (e.g. *F*, *t*, *r*) with confidence intervals, effect sizes, degrees of freedom and *P* value noted<br>*Give P values as exact values whenever suitable.*
☒	☐	For Bayesian analysis, information on the choice of priors and Markov chain Monte Carlo settings
☒	☐	For hierarchical and complex designs, identification of the appropriate level for tests and full reporting of outcomes
☒	☐	Estimates of effect sizes (e.g. Cohen's *d*, Pearson's *r*), indicating how they were calculated

Our web collection on statistics for biologists contains articles on many of the points above.

Software and code

Policy information about availability of computer code

Data collection

No software was used for collection of data.

Data analysis

- Targeted CRISPR/Cas9 DNA amplicon-seq: Reads were trimmed with trimgalore v0.4.4 to remove low-quality bases and adapters, and subsequently aligned to the human reference genome build hg19 with BBMap v34.92 allowing for 1000 bp indels. Mutations introduced by genome editing were analyzed and visualized using CRISPResso2 v2.0.27.

- ChIP-seq: ChIP-seq reads were aligned to the human reference genome build hg19 with bowtie v1.1.1 and bigwig files were generated for visualization with the bamCoverage tool from deepTools v3.4.3, with the options --normalizeUsing RPKM --smoothLength 100 --binSize 20. Peak calling was performed with MACS2 v2.2.7.1 using default settings.

- ATAC-seq: ATAC-seq reads were aligned to the human reference genome build hg19 with bowtie2 v2.3.4.1, allowing for a maximum 2000 bp insert size. Mitochondrial reads and fragments with mapping quality below 10 were removed. Bigwig files were generated as described for ChIP-seq.

- 4C-seq: Reads were aligned to the human reference genome build hg19 with Bowtie v1.1.1. Generated BAM-files were transformed into WIG-files for further processing using a custom tool. A running mean (window size 21) was applied for signal smoothing of peaks.

- 3q-Capture: Reads were aligned against hg19 using the Burrows-Wheeler Aligner v0.7.17. All chromosomal aberrations, such as translocations and inversions, were determined with BreakDancer v1.1.

- RNA-seq: Salmon v0.13.1 was used to quantify expression of individual transcripts, which were subsequently aggregated to estimate gene-level abundances with tximport. Both gene- and transcript-level abundances were normalized to counts per million (CPM) for visualization in the figures of this paper. Differential gene expression analysis of count estimates from Salmon was performed with DEseq2.

- FACS: data were analyzed with FACS Diva v9.0 and Flow Jo v10.0.

- Other: GraphPad Prism 8 was used for data visualization and statistical testing. Aligned sequencing data were visualized and compared using the Integrateve Genomics Viewer (IGV) v2.8. CTCF motifs were identified using the CTCFBSDB 2.0 database and motif orientation was retrieved from the JASPAR database (release 2020).

For manuscripts utilizing custom algorithms or software that are central to the research but not yet described in published literature, software must be made available to editors and reviewers. We strongly encourage code deposition in a community repository (e.g. GitHub). See the Nature Research guidelines for submitting code & software for further information.

Data

Policy information about availability of data

All manuscripts must include a data availability statement. This statement should provide the following information, where applicable:
- Accession codes, unique identifiers, or web links for publicly available datasets
- A list of figures that have associated raw data
- A description of any restrictions on data availability

Cell line data were stored at ArrayExpress:
E-MTAB-9958: 4C-seq of a K562 cell line model with a patient-based t(3;8) translocation
E-MTAB-9965: ChIP-seq of a K562 cell line model with a patient-based t(3;8) translocation
E-MTAB-9937: Amplicon sequencing of a K562 cell line model with a patient-based t(3;8) translocation following CRISPR treatment
E-MTAB-10785: ATAC-seq of a K562 cell line model with a patient-based t(3;8) translocation

Human data were stored at European Genome-phenome Archive (EGA):
EGAS00001004808: CTCF-dependent enhancer hijacking by the EVI1 oncogene in leukemia

Field-specific reporting

Please select the one below that is the best fit for your research. If you are not sure, read the appropriate sections before making your selection.

[x] Life sciences [ ] Behavioural & social sciences [ ] Ecological, evolutionary & environmental sciences

For a reference copy of the document with all sections, see nature.com/documents/nr-reporting-summary-flat.pdf

Life sciences study design

All studies must disclose on these points even when the disclosure is negative.

Sample size	We collected data from 57 AML patients with 3q26-rearrangements, including 10 patients with a t(3;8). We used data from all available patients with these chromosomal aberrations, which was sufficient to generate statistically significant results. We generated two parental EVI1-eGFP K562 clones and 4 t(3;8) EVI1-eGFP K562 clones. For the MUTZ-3 EVI1-eGFP line only one clone could be generated since homologue recombination in this cell line is very inefficient.<br>No sample size calculation was performed, the sample size was chosen/limited by the availability of biological material of the patient samples or generation of clones with the correct genotype.
Data exclusions	No data was excluded from analysis.
Replication	We generated two parental EVI1-eGFP K562 clones and 4 t(3;8) EVI1-eGFP K562 clones. All experiments were independently conducted and reproduced multiple times, in multiple clones as described in the main result text, the figure legends and in the main text in the Method section: Reproducibility and Statistics as requested by the editor.
Randomization	Randomization was not applicable to the current study because patients or cell lines with a t(3;8) were compared to those without a translocation. However, in experiments involving primary patient material in the control group without a t(3;8) were randomly selected.<br>In experiments involving the K562 clones, several controls were used to ensure the robustness of the conclusions.
Blinding	Blinding was not applicable to this study for all but the FISH analysis. Experimental variables required knowledge of patient or cell line genotype and importantly results were only obtained by machines (Flow cytometry, PCR, Western blot, NGS, SNP array) and not by human eye. However, appropriate controls were included in all experiments.<br>Blinding was applicable to FISH analysis of the K562 clones, this was done blind/without knowledge of intended chromosomal rearrangements by the technicians of the Clinical genetics diagnostics department of the Erasmus MC. All FISH analysis, for each clone, was done blind by two independent technicians.

Reporting for specific materials, systems and methods

We require information from authors about some types of materials, experimental systems and methods used in many studies. Here, indicate whether each material, system or method listed is relevant to your study. If you are not sure if a list item applies to your research, read the appropriate section before selecting a response.

Materials & experimental systems

n/a	Involved in the study
☐	☒ Antibodies
☐	☒ Eukaryotic cell lines
☒	☐ Palaeontology and archaeology
☒	☐ Animals and other organisms
☐	☒ Human research participants
☒	☐ Clinical data
☒	☐ Dual use research of concern

Methods

n/a	Involved in the study
☐	☒ ChIP-seq
☐	☒ Flow cytometry
☒	☐ MRI-based neuroimaging

Antibodies

Antibodies used

H3K27Ac (Diagenode C15410196),
Diagenode
H3K27Ac Antibody – ChIP-seq Grade
Polyclonal
Art. Nr: C15410196
Lot: A1723-0041D
Used: 2.84 ug/ul

H3K9Ac (Diagenode C15410004),
Diagenode
H3K9Ac Antibody – ChIP-seq Grade
Polyclonal
Art. Nr: C15410004
Lot: A1435-0012D
Used: 3.7 ug/ul

H3K4me3 (Diagenode C15410003),
Diagenode
H3K4me4 Antibody – ChIP-seq Grade
Polyclonal
Art. Nr: C15410003
Lot: A1052D
Used: 1.4 ug/ul

CTCF (Cell Signaling, 2899S),
Cell Signaling
CTCF Rabbit Ab
Polyclonal
Art. Nr: 2899S
Lot: 2
Ref: 08/2017
Used: 4ug/ul

RUNX1 (Abcam, ab23980)
Abcam: Anti-RUNX1/AML1 antibody
Art. Nr: ab23980
Lot: GR3213439-1
Used: 5 ug/IP (1mg/ml stocksolution)

EVI1 (Cell Signaling, #2265S)
Cell Signaling
Art. Nr: #2265S
Lot: 4
Ref: 04/2020
Dilution: 1:1000

B-Actin (Sigma, #A5441)
Sigma
Clone AC15
Art. Nr: #A5441
Lot: 029m48831
Dilution: 1.10.000

Validation | Validation of the antibodies directed against EVI1 and B-actin were done by Western blot. The bands of the proteins of interest were detected at the expected size in kDa as also described by the manufacturer of the antibody.
EVI1: https://en.cellsignal.jp/products/primary-antibodies/evi-1-antibody/2265?_=1608817744743&Ntt=evi1&tahead=true
B-actin: https://www.sigmaaldrich.com/catalog/product/sigma/a5441?ang=en&region=NL&gclid=Cj0KCQiAlZH_BRCgARIsAAZHSBkCSFj2FPz5x2XgrfPbUkvFvc1iXjm6KwWGA8cIRVGEN5WLFa5DXiUaAi3fEALw_wcB

Validation of the antibodies directed against H3K27ac, H3K9ac, H3K4me3 and CTCF were done by ChIP-seq, peaks were found at places that can be expected and described by the manufacturer.
H3K27ac: https://www.diagenode.com/en/p/h3k27ac-polyclonal-antibody-premium-50-mg-18-ml
H3K9ac: https://www.diagenode.com/en/p/h3k9ac-polyclonal-antibody-classic-50-ug-37-ul
H3K4me3: https://www.diagenode.com/en/documents/datasheet-h3k4me3-C15410003
CTCF: https://www.cellsignal.com/products/primary-antibodies/ctcf-antibody/2899
RUNX1: https://www.abcam.com/runx1--aml1-antibody-ab23980.html

Eukaryotic cell lines

Policy information about cell lines

Cell line source(s): The original cell line K562 (acc 10) and MUTZ-3 (acc 295) were obtained from DSMZ, the cell line MUTZ-3 EVI1-eGFP (Smeenk et al. 2021) and K562 EVI1-eGFP, K562 EVI1-eGFP t(3;8), K562 EVI1-eGFP 3q/EVI1 amplification clones were generated in house.

Authentication: Cell line audenticated by SNP array.

Mycoplasma contamination: All cell lines were tested negative, mycoplasm test were performed at a regular basis during this study.

Commonly misidentified lines (See ICLAC register): No commonly misidentified lines were used in this study.

Human research participants

Policy information about studies involving human research participants

Population characteristics: Patient samples were selected based on cytogenetic abnormalities involving 3q26, namely translocations t(3;8)(q26;q24), t(2;3)(p21;q26), t(3;7)(q26;q24) or t(3;6)(q26;q11). Of these patients 41% were male and 60% female, with an age ranging between 22-85 years of age, with an average age of 63.4 years.

Recruitment: AML patient samples were collected either from the Erasmus MC Hematology department biobank (Rotterdam, The Netherlands) or from the MLL Munich Leukemia Laboratory biobank (Munich, Germany). Patients in the t(3;8) group were selected based on cytogenetics, whereas patients in the control group were randomly selected. We are not aware of any (self select) biases that could affect the outcome of the study.

Ethics oversight: All patients provided written informed consent in accordance with the Declaration of Helsinki. The Medical Ethical Committee of the Erasmus MC has approved usage of the patient rest material for this study. The Medical Ethical Committee (Medisch Ethische ToetsingsCommissie Erasmus M, https://www.erasmusmc.nl/nl-nl/pages/metcC) of the Erasmus MC has approved usage of the patient rest material for this study.

Note that full information on the approval of the study protocol must also be provided in the manuscript.

ChIP-seq

Data deposition

- [x] Confirm that both raw and final processed data have been deposited in a public database such as GEO.
- [x] Confirm that you have deposited or provided access to graph files (e.g. BED files) for the called peaks.

Data access links (*May remain private before publication.*): https://www.ebi.ac.uk/arrayexpress/experiments/E-MTAB-9965/

Files in database submission: All raw sequencing data files are deposited in public databases and can be accessed using the numbers links provided in this form. Peak files (in BED format) are also available at the same repository.

Genome browser session (e.g. UCSC): A genome browser session can be created and provided upon request.

Methodology

Replicates: We generated two parental EVI1-eGFP K562 clones and 4 t(3;8) EVI1-eGFP K562 clones. The sepate clones have been used as biological replicates. For the patient sample derived ChIP-seq data we show no replicates, these are 3 unique AML samples.

Sequencing depth: H3K9ac ChIP libraries were sequenced paired-end with a read length of 100 bp. The average depth was 82642808 reads (SD =

13839016), including multi-mapping reads. No reads were removed after the alignment.

H3K27ac ChIP libraries were sequenced single-end with a read length of 50 bp. The average depth was 33819848 reads (SD = 1204218), excluding multi-mapping reads. No reads were removed after the alignment.

H3K4me3 ChIP libraries were sequenced single-end with a read length of 50 bp. The average depth was 33993541 (SD = 4722826), excluding multi-mapping reads. No reads were removed after the alignment.

CTCF ChIP libraries were sequenced paired-end with a read length of 100 bp. The average depth was 142327017 (SD = 24658872), including multi-mapping reads. No reads were removed after the alignment.

Antibodies

H3K27ac Diagenode #C15410196
H3K9ac Diagenode #C15410004
H3K4me3 Diagenode #C15410003
CTCF Cell signalling 2899S
RUNX1 Abcam ab23980

Peak calling parameters

Peaks were called using MACS2 callpeak v2.2.7.1, keeping all duplicates and using the human genome option. For single-end samples, BAM was specified as file type, wheras BAMPE was used for paired-end samples.

Data quality

ChIP-seq data quality was evaluated as per ENCODE standards (Landt S et al, Genome Res 2012). Namely, for each sample we considered the following parameters: total depth, number of peaks, fraction of reads in peaks (FRIP), normalized strand coefficient (NSC) and relative strand correlation (RSC). All the samples analyzed here complied with the ENCODE standards.

Software

ChIP-seq reads were aligned to the human reference genome build hg19 with bowtie and bigwig files were generated for visualization with bedtools genomecov and UCSC bedGraphToBigWig. Peak calling was performed with MACS2 using default settings. Quality metrics were computed with an in-house algorithm.

Flow Cytometry

Plots

Confirm that:

- [x] The axis labels state the marker and fluorochrome used (e.g. CD4-FITC).
- [x] The axis scales are clearly visible. Include numbers along axes only for bottom left plot of group (a 'group' is an analysis of identical markers).
- [ ] All plots are contour plots with outliers or pseudocolor plots.
- [ ] A numerical value for number of cells or percentage (with statistics) is provided.

Methodology

Sample preparation

All Flow Cytometry was done on K562 clones. Cells were measured alive in FACS buffer.

Instrument

FACS Canto or the FACS Aria flow cytometer (BD Biosciences)

Software

FACS Diva version 9.0 and Flow Jo version 10.

Cell population abundance

All experiments were done on a single clone derived cell population.

Gating strategy

Viable cells were gated in the FCS-A SSC-A plot, followed by dublicate exclusion in the FCS-A FCS-H plot before analysing eGFP levels of the different clones or experimental samples in the FIT-C channel. eGFP negativity or positivity was gated by the use of the eGFP negative WT K562 cells, compared to the corresponding parental EVI1-eGFP clones or to the eGFP levels in the cell population of the control experiment done in the same K562 t(3;8) EVI1-eGFP clone. For each measurement the corresponding correct control was measured in the same experiment.

- [x] Tick this box to confirm that a figure exemplifying the gating strategy is provided in the Supplementary Information.
